# Glycosides from edible sea cucumbers stimulate macrophages *via* purinergic receptors

**DOI:** 10.1038/srep39683

**Published:** 2016-12-22

**Authors:** Dmitry Aminin, Evgeny Pislyagin, Maxim Astashev, Andrey Es’kov, Valery Kozhemyako, Sergei Avilov, Elena Zelepuga, Ekaterina Yurchenko, Leonid Kaluzhskiy, Emma Kozlovskaya, Alexis Ivanov, Valentin Stonik

**Affiliations:** 1 G.B. Elyakov Pacific Institute of Bioorganic Chemistry, Far Eastern Branch of the Russian Academy of Sciences, Vladivostok, 690022, Russia; 2 Institute of Cell Biophysics, Russian Academy of Sciences, Pushchino, Moscow Region 142290, Russia; 3 Institute of Biomedical Chemistry, Moscow, 119121, Russia

## Abstract

Since ancient times, edible sea cucumbers have been considered a jewel of the seabed and used in Asian folk medicine for stimulation of resistance against different diseases. However, the power of this sea food has not been established on a molecular level. A particular group of triterpene glycosides was found to be characteristic metabolites of the animals, responsible for this biological action. Using one of them, cucumarioside A_2_-2 (CA_2_-2) from the edible *Cucumaria japonica* species as an example as well as inhibitory analysis, patch-clamp on single macrophages, small interfering RNA technique, immunoblotting, SPR analysis, computer modeling and other methods, we demonstrate low doses of CA_2_-2 specifically to interact with P2X receptors (predominantly P2X4) on membranes of mature macrophages, enhancing the reversible ATP-dependent Ca^2+^ intake and recovering Ca^2+^ transport at inactivation of these receptors. As result, interaction of glycosides of this type with P2X receptors leads to activation of cellular immunity.

Edible sea cucumbers (class Holothurioidea, phylum Echinodermata) are exclusively marine animals, foraged for centuries as one of the most valuable kind of sea foods in different geographical regions from Asia to Oceania and Australia. Being harvested for consumption (about 160,000 tonnes annually, FAO FishStat database), these creatures, known as *bêches de mer* or *trepangs,* are considered not only a delicacy in China, Korea, Japan and other Asian countries (from the recent time this food became to be in great request in USA, Russia and European countries also), but also they are widely used in traditional Asian folk medicine. The corresponding preparations from sea cucumbers are recommended for protection and treatment of different ailments, including infections, cancer, and arthritis. However, scientific evidence to support their efficacy and all the more so information concerning the molecular mechanisms of action of natural compounds characteristic of this sea food are scanty.

A particular group of secondary metabolites, so-called holostane triterpene glycosides, were found exclusively in these invertebrates and some sea cucumber-eating starfish. Cucumarioside A_2_-2 (CA_2_-2) from the Far-Eastern edible sea cucumber *Cucumaria japonica* (Fig. 1, Supplementary Fig. S1), one of 100 characteristic and related to each other sea cucumber triterpene glycosides isolated by our group from 60 sea cucumber species (Supplementary Table S1), was selected to study the molecular mechanism of the immunostimulatory action at peroral application of these metabolites, including the previously described activation of macrophages^1^,^2^,^3^,^4^ and enhancing antibacterial resistance *in vivo*^5^. In previous studies, it has been shown that at nanomolar doses, CA_2_-2 stimulates adhesion, spreading, motility of macrophages^3^, and increases the cytoplasmic Ca^2+^ level^2^,^6^. Besides immunostimulatory action, this and other related sea cucumber glycosides show antitumor, anti-radiation and other useful properties^7^,^8^,^9^,^10^,^11^,^12^,^13^,^14^.

In this study, we focus on the search for molecular targets of CA_2_-2 and on detailed investigation of its interaction with the targets on membrane of murine peritoneal macrophages, using Ca^2+^-imaging, confocal microscopy, flow cytometry, inhibitory analysis, blocking the receptors by the corresponding antibodies, knockdown experiments, electrophysiology, Western blotting, SPR analysis and computer modeling. Herein, we report that immunostimulatory action of this characteristic of sea cucumbers glycoside is a result of its interaction with macrophageal P2X family receptors (predominantly P2X4) and induction of ATP-dependent reversible Ca^2+^ signaling followed by the recovering the receptor’s inactivation.

## Results

### CA_2_-2 induces a reversible [Ca^2+^]_i_ increase in macrophages via P2X receptors

**Figure 1:**
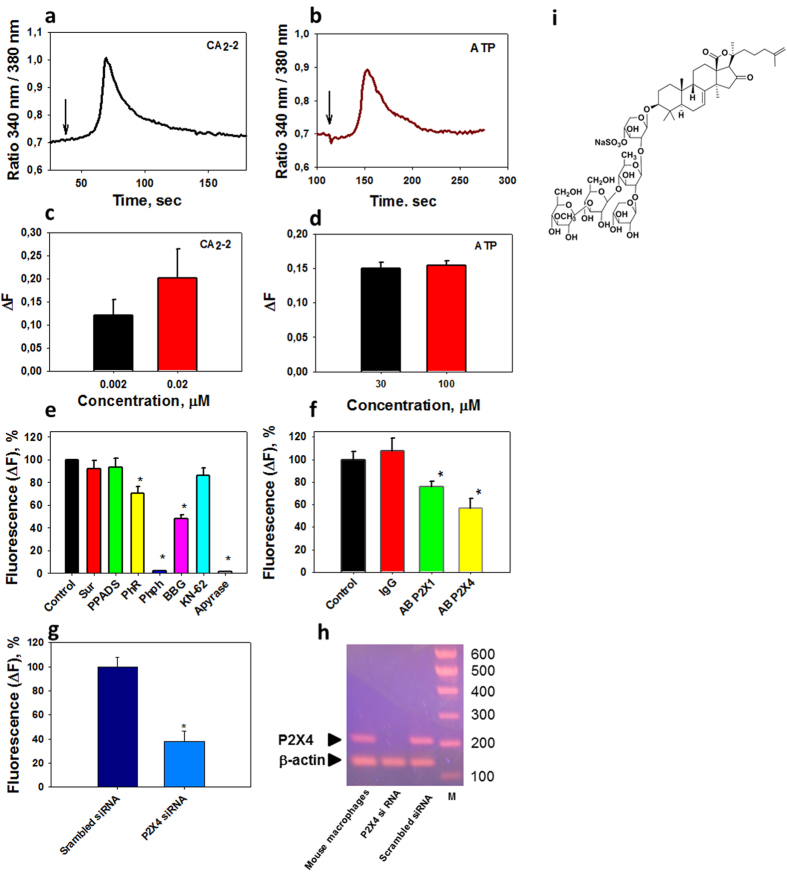
CA_2_-2 at nanomolar concentrations induces a reversible [Ca^2+^]_i_ increase in macrophages by binding with purinergic receptors of the P2X family. (**a,b**) Influence of CA_2_-2 (0.02 μM) or ATP (30 μM) on duration and (**c,d**) amplitude of Ca^2+^ response in murine peritoneal macrophages, loaded with Ca^2+^-sensitive fluorescent probe Fura-2/AM. Arrows show the moment of addition of inductors to macrophages. (**e**) Pre-incubation with different calcium channel blockers and apyrase followed by Ca^+2^ influx at application of CA_2_-2: 1 control; 2 suramin, 100 μM; 3 PPADS, 10 μM; 4 PhR 10 μM; 5 Phph, 10 μM; 6 BBG 100 μM; 7 KN-62, 10 μM; apyrase, 2.0 U. (**f**) Effect of 30 min pre-incubation with receptor antibodies (ab81122, rabbit polyclonal to P2X1, Abcam,10 μg/mL, orb100036, rabbit polyclonal to P2X4, Biorbyt, 10 μg/mL) and normal rabbit immunoglobulin G (IgG, 10 μg/mL) on Са^2+^ influx into peritoneal macrophages (mice, Balb/с line), caused by CA_2_-2 (100 nM); cells alone were used as a control. (**g**) Effect of transient knockdown approach, using P2X4 small interfering RNA, confirms a significant role for the P2X4 receptor in conferring CA_2_-2 induced Ca^2+^ entry. (**h**) Expression of P2X4 mRNA in mouse macrophages and reduction of P2X4 mRNA in macrophages by P2X4 siRNA treatment in comparison with macrophages treated with scrambled RNA (PCR data). In addition, control reactions for loading were performed for each sample using β-actin specific primers generating the expected product. DNA molecular weight markers (M) are indicated in base pairs (bp). (**i**) chemical structure of CA_2_-2. All data are presented as m ± sd, *p < 0.05.

The first objectives of the study were to establish the reasoning behind Ca^2+^ spikes in approximately 20–30% of murine peritoneal macrophages (Supplementary Video S1 and Supplementary Fig. S2a, b), including corresponding molecular targets. Using a Ca^2+^ imaging technique, we have shown that the application of either ATP or CA_2_-2 to a monolayer of macrophages induces a sharp reversible increase in cytoplasmic Ca^2+^ concentration. We found that CA_2_-2 was approximately 1000 times more potent than ATP itself (Fig. 1a–d) in induction of calcium oscillations as means to transmit the biological information from the chemical stimulus to macrophages. It is known that ATP causes the penetration of Ca^2+^ into macrophages from the external environment by selective interaction with P2X family receptors in their plasmatic membranes. Earlier, a limited set of purine receptors of the P2X family, namely P2X1, P2X4 and possibly P2X7, were found in the macrophageal membrane^15^.

To study whether the same receptors participate in the calcium signaling at CA_2_-2 treatment, we have examined the influence of different P2X receptor blockers on macrophages, followed by CA_2_-2 application provoking the reversible Ca^2+^ influx (Fig. 1e). However, P2X1 blockers, Sur (suramin) and PPADS (pyridoxal-5-phosphate-6-azophenyl-2′,4′-disulphonic acid) did not inhibit Ca^2+^ input. KN-62 (1-[N,O-bis(5-isoquinolinesulfonyl)-N-methyl-L-tyrosyl]-4-phenylpiperazine), a selective blocker of the P2X7 receptor, also did not induce notable changes in Са^2+^ transport. When we used phenol red (PhR), another Р2Х1 blocker, there was approximately 30% inhibition of Са^2+^ influx into cells. Using phenolphthalein (Phph), which is highly selective to P2X4 receptors, and Brilliant blue G-250 (BBG), which is selective against both P2X4 and P2X7 receptors, we inhibited the effect of CA_2_-2 on 100% and 50%, respectively. Thus, the inhibitory analysis has shown some receptors of the Р2Х family (predominantly P2X4) might have been molecular targets of the glycoside applied to macrophages at super low doses.

The pretreatment of macrophages with apyrase, an enzyme that cleaves and eliminates ATP from the incubation medium, inhibited the CA_2_-2 activating effect on Ca^2+^ entry almost completely (Fig. 1e). These data confirmed that purinergic receptors, belonging to the P2 class, which response to the release of ATP, are the molecular targets of CA_2_-2 in plasmatic membrane of macrophages, and that action of the glycoside is ATP-dependent.

Partial inhibition of the effect of CA_2_-2 on macrophages at pretreatment with P2X1- and particularly with P2X4-antibodies (Fig. 1f), hampering its interaction with these receptors, also corresponded the suggestion about P2X4-receptors as main molecular targets of this natural product, isolated from sea cucumbers.

To examine further the role of expressed in mouse macrophages P2X4 receptors in the CA_2_-2-induced Ca^2+^ entry molecular mechanism, cells were transiently transfected with the effective P2X4 siRNA. Compared with scrambled siRNA transfected controls, the transient transfection with P2X4 siRNA constructs inhibited the Ca^2+^ influx induced by glycoside in more than two times (Fig. 1g). Simultaneously, silencing RNA interference caused the significant knockdown of P2X4 mRNA expression in macrophages as was shown by PCR (Fig. 1h). Together, these data confirm a crucial role of P2X4 receptors in [Ca^2+^]_i_ increase in CA_2_-2-stimulated macrophages.

### CA_2_-2 interacts mainly with mature macrophages enriched for P2X receptors

To understand why only a part of the total population of mouse peritoneal macrophages responds to CA_2_-2 treatment, we used flow cytometry and image analysis. Two main subpopulations (small ungranulated spherical cells with an average diameter of 7.13 ± 0.88 μm, 60–65%, and larger cells with a diameter of 12.77 ± 2.20 μm and rough surface, 25–30%) were brought into light (Supplementary Fig. S3).

**Figure 2:**
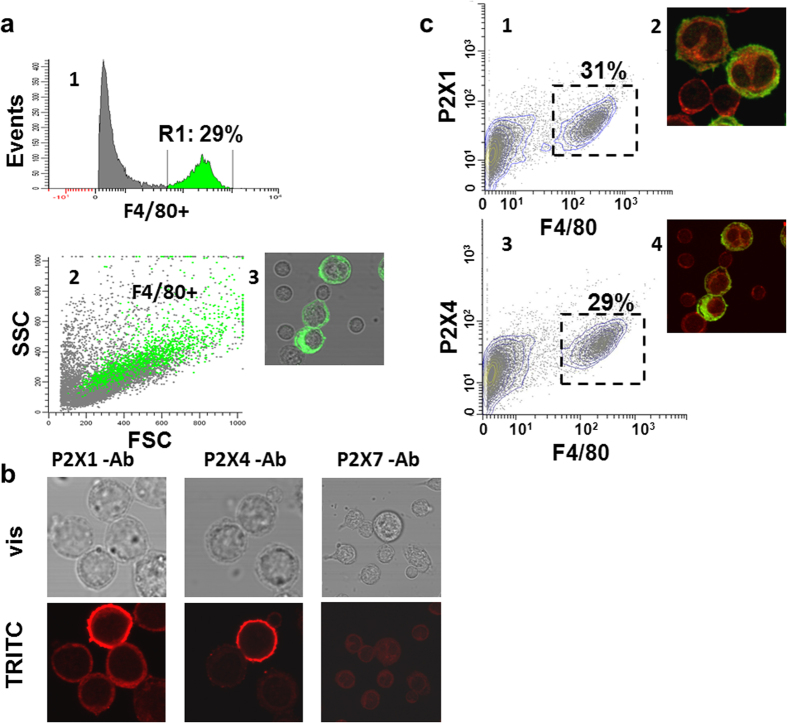
P2X receptors, the main molecular targets of CA_2_-2, are localized on membranes of large granular macrophages (mature macrophages). (**a**) Percentage of F4/80+ cells in a total fraction of peritoneal BALB/c mouse macrophages (mature macrophages) determined by flow cytometry (1, 2) and confocal microscopy (3). Mature macrophages were dyed green on a dotogram (2) and in the confocal representation (3). (**b**) Localization of P2X receptors on the surface of peritoneal macrophages from BALB/c mice by the immunocytochemical method followed by confocal microscopy. Secondary antibodies were conjugated with TRITC (red staining). (**c**) Colocalization of Р2Х+ and F4/80+ cells in a population of peritoneal mouse macrophages, indicated by flow cytometry (1, 3) and confocal microscopy (2, 4). Immunochemical staining of the cells using antibodies to F4/80 and FITC (green staining) and to Р2Х1, P2X4, and TRITC (red staining).

Staining with antibodies to the mature macrophage marker F4/80 (Supplementary Fig. S3) along with flow cytometry and confocal microscopy, showed that F4/80 + cells represent the large macrophages (Fig. 2a). Immunocytochemical staining with antibodies to the P2X1 and P2X4 receptors indicated these receptors on mature macrophage surfaces, while the P2X7 receptor type was detected there only in trace amounts (Fig. 2b). The percentage of macrophages enriched for P2X1 and P2X4 receptors was found to be approximately 30% (Supplementary Fig. S5).

Histogram analysis confirmed that a high density of P2X1 and P2X4 receptors (mature macrophages) occurs in approximately 30% of macrophages, while smaller sized cells contain fewer receptors of the P2X family, with the exception of P2X7 receptors (Fig. 2c; Supplementary Fig. S4). This suggests that CA_2_-2 interacts mainly with mature macrophages enriched for P2X receptors. Colocalization of Р2Х+ and F4/80+ cells was confirmed using antibodies to Р2Х1 and Р2Х4 receptors and TRITC as a fluorescent probe, as well as antibodies to F4/80+ and FITC.

### CA_2_-2 recovers the sensitivity of inactivated P2X receptors to ATP

**Figure 3:**
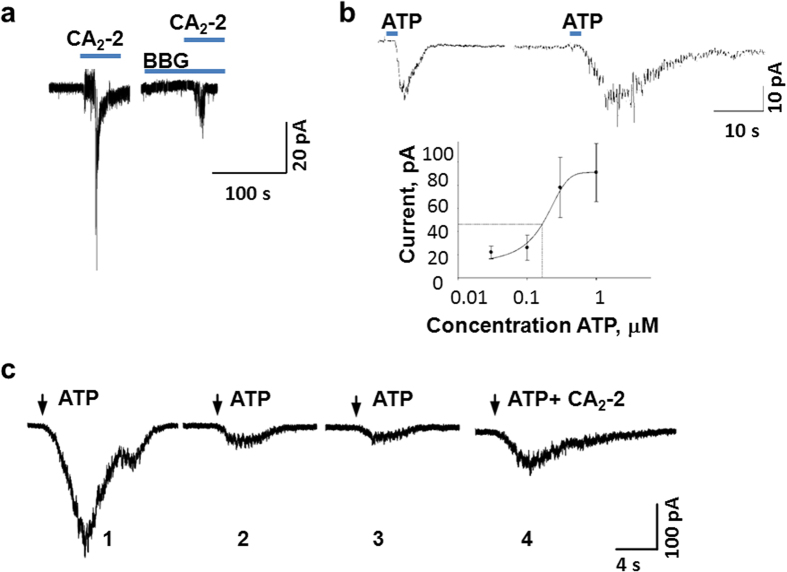
CA_2_-2 increases the Ca^2+^ current in single macrophages as a result of action on P2X family receptors and partly recovers the inactivation of P2X receptors caused by repeated application of ATP. (**a**), Patch clamp in configuration of “whole-cell”. Registration of Са^2+^ current in single macrophage at application of CA_2_-2 (100 nM) before and after pre-treatment with BBG (100 μM). (**b**) Registration of Са^2+^ current evoked by ATP, and ATP after pre-treatment with CA_2_-2. Left, membrane potential −40 mV, ATP, 0.03 μМ, 2 sec, on the right, ATP, 0.03 μМ, 2 sec after 2 min pre-incubation with CA_2_-2, 100 nM, below a concentration–response relationship for ATP, EC_50_ = 0.16 μМ. (**c**) The clearing of inactivation of single macrophage by CА_2_-2, 1-3 – consequent answers of the cell to action of repeated portions of 0.3 μМ of ATP, and 4 – after 0.3 μМ ATP + 100 nM CA_2_-2 with 2 minutes headway. Cells that were not pretreated with apyrase (**a**) and pretreated with apyrase (**b,c**).

To argue additionally that the glycoside interacts with P2X receptors and assess how this interaction is connected with ATP action on Са^2+^ transport, we used patch-clamp on single macrophages. The response of macrophages that were not pre-incubated with apyrase to ATP was detected at the application of as high doses as 0.1–1.0 mM. Some cells did not respond, while others suffered from the rapidly increased leakage current and were unable to sustain more than one application of ATP (Supplementary Fig. S6). The treatment with CA_2_-2 (100 nM) elicited a sharp incoming calcium current under the same conditions. Furthermore, the pre-incubation of the cells with BBG blocker selective for P2X4 and P2X1 receptors led to effective inhibition of a current evoked by CA_2_-2 (Fig. 3a), confirming the participation of the receptors in the action of CA_2_-2. In addition, macrophages pre-incubated with apyrase were proved to be much more sensitive to the action of ATP (EC_50_ = 0.16 μM)^16^ (Supplementary Fig. S5).

CA_2_-2 alone did not influence the membrane conductivity of apyrase-treated cells. However the pretreatment with CA_2_-2 significantly increased the response of macrophages to ATP. In fact, when ATP was applied immediately after this treatment, some peculiarities of the Ca^2+^ current were altered, including current amplitude (∼56% increase), at that total number of Ca^2+^ ions, penetrated into the cell was increased 4-fold when compared with the effect of ATP alone (Fig. 3b). These results are similar to those obtained using Ca^2+^ imaging (Supplementary Fig. S6).

Repeated ATP applications to macrophages induced the successive desensitization of receptors up to their complete inactivation after three consecutive applications. The application of CA_2_-2 after three treatments with ATP partly recovered the sensitivity of these cells to ATP (Fig. 3c). Therefore, this sea food constituent is capable not only of enhancing the cell’s response to ATP, but also of reversing receptor desensitization and recovering the Ca^2+^ conductivity of the macrophageal membrane.

### CA_2_-2 and ATP have the different binding sites localized on the P2X receptor

**Figure 4:**
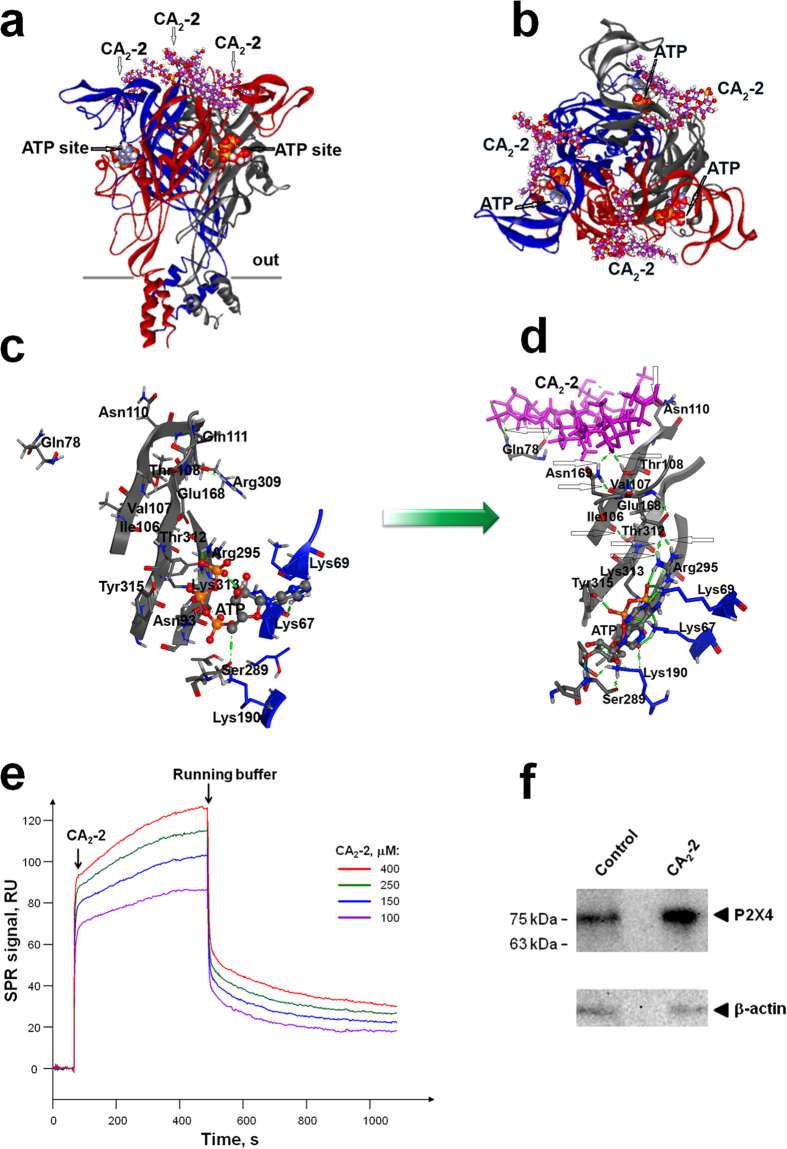
The interaction of CA_2_-2 with P2X4 receptor. (**a)** (side view), (**b)** (top view), The modeling of the mP2X4 receptor (30-354 amino acid residues) with ATP and CA_2_-2 interaction is shown. Membrane surface is grey, different subunits of the receptor are red, blue and grey, ATP is presented as sphere packing structure, CA_2_-2 as ball-and-stick diagram by magenta with oxygen atoms marked by red, hydrogen atoms marked by white and sulfur atoms marked by yellow. Binding sites of ATP and CA_2_-2 are shown by arrows. (**c,d)** Fragments of mP2X4 receptors, containing β-sheets with localized ATP and CA_2_-2 molecules in initial and modified by CA_2_-2 states, respectively. ATP is shown by ball-and-sticks, CA_2_-2 by magenta sticks, amino acids residues, interacting with the ligands by grey and blue in dependence on the belonging to some or other receptor. Green arrow indicates the changes in structure of mP2X4 receptor after interaction with CA_2_-2. (**e)** Sensograms of CA_2_-2 (100-150-250 and 400 μM) interaction with immobilized Р2Х4 receptor. The arrows indicate start of the CA_2_-2 injections and the end of CA_2_-2 injections, and start of complex dissociation during washing with the running buffer. (**f)**, P2X4 protein expression in mouse peritoneal macrophages in control cells and after stimulation of mouse peritoneal macrophages with CA_2_-2 (10 nM) for 48 h. Western blot of total P2X4 receptors and β-actin from macrophages are shown.

To get outside of the receptor-binding events during interaction of CА_2_-2 with P2X4, we used spatial structures of the zfP2X4 receptor and its complex with ATP, established by X-ray analysis^17^,^18^ and carried out *in silico* modeling by so-called “blind molecular docking” of CA_2_-2 and ATP over the rigid structure of the extracellular domain of the receptor. Three-dimensional structures of mP2X + ATP, mP2X + CA_2_-2 and mP2X + ATP + CA_2_-2 complexes were generated. The ATP-binding site was shown to be localized with close contacts to amino acid residues Lys67, Lys69, Glu84, Thr186, Leu187, Leu211, Lys215, His213, Thr214, Val229 of one receptor subunit, and Ser284, Ser285, Asn288 Arg298, Arg295 and Lys313 of another subunit (numbering corresponds to the amino acid sequence of mP2X4). This is in agreement with experimental data earlier obtained from studies on the zfP2X4 receptor with and without ATP^19^. The binding site of CA_2_-2 on the extracellular moiety of the mP2X4 receptor does not overlap the ATP-binding site (Fig. 4a–d), indicating a lack of competition of both ligands for their binding sites.

According to molecular dynamics calculations, Asn75, Ser77, Gln78, Leu79, Gly80, Asn110, Val109, Pro166, Phe152, Val167, Pro117, Thr108 and Asn169 of one subunit, and Arg301, Ala304 and Gly305 amino acid residues of another subunit participate in CA_2_-2 binding with the formation of hydrogen bonds with some of these residues (Fig. 4; Supplementary Fig. S7). Taking into account the contribution of Gln78, Asn110, Thr108, and Asn169 in the interaction of the ligand with the receptor, the free energy of CA_2_-2 binding with mP2X4 was calculated to be of 13.7 kcal/mol (Supplementary Table S2).

To confirm the interaction between CA_2_-2 and P2X4 receptors a surface plasmon resonance (SPR) analysis was carried out. For this purpose the mouse recombinant P2X4 receptors were immobilized on the optical biosensor chip of Biacore T200 with the effectiveness of about 3.3 ng Р2Х4 protein/mm^2^. The direct binding of CA_2_-2 with P2X4 receptors was detected and Kd value for the complex of Р2Х4*/*CA_2_-2 was calculated as 45.2 × 10^−6^ M. The dose-response curves of SPR kinetic analysis are shown on Fig. 4e.

We examined the effect of CA_2_-2 on the protein expression level of P2X4 receptor family in mouse peritoneal macrophages. Western blot analysis of total cellular protein preparations with P2X4-specific antibodies (Fig. 4f) revealed one main immunoreactive band of around 75 kDa, corresponding to the glycosylated form of this subunit. We found that the P2X4 receptor expression was visibly enhanced in macrophages incubated with CA_2_-2 which reflects the stimulatory effect of triterpene glycoside on immune cells.

## Discussion

It is well known that calcium signaling governs many cellular processes^20^, including the activation of macrophages, while calcium transport blockers inhibit this activation^21^. Cytosolic calcium oscillations present signal-specific information, which is crucial for different cell responses and influence the efficiency and specificity of gene expression^22^. Herein, we have shown the glycosides from edible sea cucumbers, such as CA_2_-2 interact with P2X family receptors (predominantly with P2X4) at low doses, inducing cytosolic calcium oscillations in mature macrophages.

Taking into attention that in accordance with structural models of mP2X4 complexes with CA_2_-2, generated by *in silico* modeling, CA_2_-2 and ATP-binding sites are localized at different areas of the extracellular receptor domain, it may be concluded that these ligands not only do not compete for interaction with macrophages, but also glycosides from sea cucumbers are potent allosteric modulators of the receptor’s activities. Since molecular dynamics simulations suggested that the CA_2_-2 action on these receptors turns them into binding sites with the formation of an H-bond network, the creation of such network between the two binding sites probably determines the nature of CA_2_-2 influence on ATP interaction with P2X4 receptor and is responsible for the modulation of the receptor activity.

As a consequence of Ca^2+^ transport regulation, glycosides enhance expression of genes involved in the activation of cellular immunity. Moreover, as we have shown previously^23^, not only CA_2_-2, but also frondoside A from another edible sea cucumber *Cucumaria frondosa*, upregulate expression of Septin-2 and some other proteins, immobilizing pathogenic bacteria and preventing their invasion into human cells, thereby participating in the immune response to infections.

Therefore, the consumption of sea cucumbers followed the penetration of CA_2_-2 into blood (that was confirmed by us using MALDI mass-spectrometry) followed by the interaction of CA_2_-2 and/or related compounds with P2X receptors resulted in the stimulation of macrophages and may explain earlier established positive effects of these natural products, including increase of antibacterial resistance against pathogenic bacteria^5^, anti-radiation action^8^, the enhancement of the anticancer action of antitumor drugs^9^,^10^, etc.

Many human diseases are accompanied by the presence of ATP in the extracellular medium during inflammation. This leads to inactivation of purinergic receptors on membranes of immune cells, the inability of macrophages to response to even higher ATP concentrations, and finally, immunosuppression and the blocking of infection control^24^. CA_2_-2 application and the application of some other glycosides from sea cucumbers not only stimulate macrophageal activities, but also output a part of the P2X receptors from their inactivated state, and, as a result, the original immune response can reverse immunodeficiency (Supplementary Fig. S8).

Purinergic receptors, which now are associated with a series of important functions in normal and pathophysiology, are known as molecular targets, useful at the search for new low-molecular-weight pharmaceutical leads. For example, it was recently established the cardiac P2X4 receptors are a new cardioprotective target in heart failure^25^, the same receptors in brain present a novel target for the development of drugs to prevent and treat alcohol use disorders^26^. Our data demonstrate that macrophageal P2X4 receptors may be considered a molecular target that governs immune response against infections and some other stress factors.

## Methods

### Triterpene glycoside isolation

Monosulfated triterpene glycoside cucumarioside А_2_-2 (CA_2_-2) is 3-*O*-{3-*O*-methyl-β-D-glucopyranosyl-(1→3)-β-D-glucopyranosyl-(1→4)-[β-D-xylopyranosyl-(1→2)]-β-D-quinovopyranosyl-(1→2)−4-*O*-sodium sulfate-*β*-D-xylopyranosyl}−16-keto-holosta-7,25-diene.

Melting point (MP) 245–247 °C. [α]_D_^20^: −68 (*c* 0.1 pyridine).

^13^C-NMR (126.04 MHz, C_5_D_5_N) δ: 36.0 (C-1), 27.1 (C-2), 89.1 (C-3), 39.7 (C-4), 48.5 (C-5), 23.3 (C-6), 121.9 (C-7), 144.2 (C-8), 47.2 (C-9), 35.9 (C-10), 22.6 (C-11), 29.8 (C-12), 56.8 (C-13), 45.8 (C-14), 52.1 (C-15), 213 (C-16), 63.7 (C-17), 178.8 (C-18), 24.1 (C-19), 83.5 (C-20), 26.3 (C-21), 39.0 (C-22), 22.6 (C-23), 38.1 (C-24), 145.7 (C-25), 110.4 (C-26), 22.3 (C-27), 17.5 (C-30), 28.9 (C-31), 32.0 (C-32), 104.9 (C’-1), 81.8 (C’-2), 76.0 (C’-3), 76.3 (C’-4), 64.6 (C’-5), 102.4 (C”-1), 82.4 (C”-2), 76.0 (C”-3), 87.0 (C”-4), 71.2 (C”-5), 18.1 (C”-6), 105.1 (C”’-1), 73.8 (C”’-2), 88.0 (C”’-3), 69.8 (C”’-4), 77.9 (C”’-5), 62.1 (C”’-6), 105.6 (C””-1), 75.1 (C””-2), 88.0 (C””-3), 70.7 (C””-4), 78.4 (C””-5), 60.7 (OMe), 105.6 (C””’-1), 75.1 (C””’-2), 76.7 (C””’-3), 70.7 (C””’-4), 66.6 (C””’-5).

Cucumarioside А_2_-2 was isolated from the sea cucumber *Cucumaria japonica* by standard procedures^27^ and kindly provided by Drs Avilov S. A. and Silchenko A. S. (PIBOC FEBRAS, Vladivostok, Russia). The glycoside was individual by ^1^H and ^13^С NMR. The chemical formula of cucumarioside А_2_-2 and photograph of the sea cucumber *Cucumaria japonica* are presented in Fig. 1 and Supplementary Fig. S1, correspondingly.

### Peritoneal macrophage isolation

BALB/c mice were sacrificed by cervical dislocation. Peritoneal macrophages were isolated using standard procedures^28^. For this purpose 1 ml of PBS (pH 7.4) was immediately injected into the peritoneal cavity and the body intensively palpated for 1-2 min. Then the peritoneal fluid was aspirated with a syringe. Two hundred and fifty microliters of mouse peritoneal macrophage suspension was applied to a glass cover slips and left at 37 °C in an incubator for 1 hour to facilitate attachment of peritoneal macrophages to the dish. Then a cell monolayer was triply flushed with PBS (pH 7.4) for deleting attendant lymphocytes, fibroblasts and erythrocytes and cells were used for further image analysis or electrophysiology assay. Immunocytochemistry, Ca^2+^-signaling and whole-cell recordings were carried out at least 2 h after removal of macrophages from peritoneal cavity.

All animal experiments were conducted in compliance with all rules and international recommendations of the European Convention for the Protection of Vertebrate Animals used for experimental and other scientific purposes. All procedures were approved by the Animal Ethics Committee at the G. B. Elyakov Pacific Institute of Bioorganic Chemistry, Far Eastern Branch of the Russian Academy of Sciences (Vladivostok, Russia), according to the Laboratory Animal Welfare guidelines.

### Ca^2+^-imaging in macrophages

Ca^2+^-imaging in macrophages was performed according to standard methods^29^. Plated on coverslip macrophages were loaded with 10 μM Fura-2AM (Molecular Probes, Eugene, OR, USA) and incubated for 40 min at room temperature in saline solution: NaCl – 140 mM, KCl – 5 mM, CaCl_2_ – 1 mM, HEPES – 10 mM, pH 7.4. Cells were then washed with the same solution but without fluorescent dye and incubated for 20–30 minutes to allow the cells to recover. The coverslips were then mounted in a confocal imaging chamber (RC-30HV, Warner Instruments, Hamden, CT, USA) and the chamber was placed onto the stage of a fluorescent scanning device composed on the base of an inverted microscope (Axiovert 200, Zeiss, Oberkochen, Germany). A luminescent 75-W Optosource xenon arc lamp and a DAC-controlled Optoscan monochromator (Cairn Research Ltd., Faversham, UK) were used as light sources for inducing fluorescence and an HQ FURA filter-block (Chroma Technology Corp., Bellows Falls, VT, USA) and a Fluar 40 × /1.3 Oil objective (Zeiss) were used to visualize cell fluorescence. The chamber was perfused with saline solution by gravity at 1 ml min^−1^. Test compounds were directly introduced into the flow of saline solution. Intracellular calcium concentrations of groups of 20–40 cells were monitored continuously at room temperature with the use of a digital monochrome video camera (Orca-ER C4742-95, Hamamatsu Photonics K.K., Hamamatsu City, Japan) and an IBM-compatible Pentium-IV computer with a Firewire data interface card. Fura-2 was excited alternately at 340 and 380 nm, and the emission at 510 nm was measured with AQM Advance 6 computer program (Kinetic Imaging Ltd., Bromborough, UK). In some cases the Ca^2+^-imaging system based on Zeiss observer Z1 microscope (Zeiss, Oberkochen, Germany) with Sutter Lambda DG4 light source (Sutter Instruments, Novato, CA, USA) and Perfusion Fast-Step system SF-77B (Warner Instruments, Hamden, CT, USA) was used in Institute of Cell Biophysics, RAS. All data are expressed as the Fura-2 fluorescence 340/380 nm ratio changes against time.

Calcium ionophore ionomycin (Sigma-Aldrich, Poole, Dorset, UK) was used as a positive control to reach the maximal Ca^2+^ intracellular level. Experiments in Ca^2+^-free medium were performed in solution containing NaCl – 145 mM, glucose – 10 mM, KCl – 5 mM, MgCl_2_ – 0.8 mM, HEPES – 10 mМ, EGTA – 5 mM (pH 7.4). In some cases cells loaded with Fura-2 were pre-incubated with different P2X receptor antagonists or antibodies for 30 min before experiment.

### Whole-cell recording and agonist application

Whole-cell recording was carried out using standard method^15^. Coverslips with attached macrophages were preliminary placed in a medium (NaCl – 147 mM, D-glucose – 13 mM, KCl – 3 mM, MgCl_2_ – 1 mM, HEPES – 10 mM; pH 7.4), containing apyrase (2U ml^−1^; Apyrase from potato, Sigma-Aldrich, Poole, Dorset, UK) and incubated for 2–5 h at 37 °C. Then coverslips were placed in a recording chamber and mounted in electrophysiology chamber on the stage of a microscope. Extracellular recording solution containing (mM): NaCl, 147; KCl, 3; MgCl_2_, 1; CaCl_2_, 2; HEPES, 10 and D-glucose, 13 (pH adjusted to 7.4 with NaOH) was superfused by gravity at a rate of 1 ml min^−1^. Registration of ion currents was carried out in the condition of microelectrode contact with membrane fragments perforated with nystatin (Sigma-Aldrich, Poole, Dorset, UK). Patch electrodes and puffer electrodes were filled with an intracellular solution containing: NaCl – 145 mM, MgCl_2_ – 1 mM, HEPES – 10 mM, EGTA – 0.5 mM; pH 7.2, nystatin – 150 μg/ml. Whole-cell recordings were made at room temperature using an Model 2400 amplifier (AM Systems, USA), and data collected using L-791 interface (L-Card, Moscow, Russia) and WinWCP 3.2.6 (Strathclyde Electrophysiology Software, UK). Test compounds were applied via a glass ‘puffer’ pipette, and a change of surrounding cell solution was carried out using a pneumatic fast-step system with speed switch < 0.5 sec. Antagonists were applied by superfusion for 15 min prior to the application of ATP or glycoside (with antagonist). The ATP- or cucumarioside A_2_-2-evoked currents were then compared with those observed in other cells with no antagonist pretreatment.

### Immunocytochemistry

Macrophages on coverslips were washed once with PBS, fixed with cold 100% methanol for 5 min in ice bath, and then washed (3 × 5 min) with PBS. For F4/80 staining, cells were blocked with 10% bovine serum in PBS containing 0.1% Triton X-100 for 1 h at room temperature before incubating with rat anti-mouse F4/80 antibody or CD14 (1:200; BioLegend Inc., San Diego, CA), 5% bovine serum in PBS for 18–20 h at 4 °C. Cells were washed with PBS (3 × 5 min) and incubated with FITC-conjugated donkey anti-rat secondary antibody (1:200; BioLegend Inc., San Diego, CA) for 1 h at room temperature, then rinsed in PBS and mounted in a confocal imaging chamber for further anaysis. For P2X receptor immunocytochemistry, cells were blocked with 10% bovine serum in PBS containing 0.1% Triton X-100 (1 h) and incubated with primary antibodies for P2X1 (1:1000; Abcam plc, Cambridge, UK), P2X4 (1:1000; Biorbit Ltd., Cambridge, UK) or P2X7 1:500; Abcam plc, Cambridge, UK) overnight at 4 °C. Cells were washed with PBS (3 × 10 min) and incubated in TRITC-conjugated goat anti-rabbit secondary antibody (1:200, Abcam plc, Cambridge, UK) for 2 h at room temperature. Cells were rinsed in PBS (3 × 10 min) before mounting. Negative controls were performed by omitting the primary antibodies. Examination of macrophage fluorescence was performed with an LSM 510 META confocal laser scanning microscope (Carl Zeiss, Gottingen, Germany) equipped with an Ar laser with an effective power of 30 mW. Acquired images were analyzed with LSM510 Release 3.5 software (Carl Zeiss, Gottingen, Germany). The suspension of macrophages was fixed and stained with antibodies against P2X1 and P2X4 receptors as described above, and then analyzed with fluorescent flow cytometer FACScalibur (Becton-Dickinson, USA). Data acquisition and estimation of results were performed using BD CellQuest Pro (Becton-Dickinson, USA) and WinMDI 2.9 (USA).

### Western blot

The expression of P2X4 was studied by protein electrophoresis and Western blotting methods. Mouse peritoneal macrophages were cultured in DMEM culture medium supplemented with 100 μg/ml gentamicin in the presence of CA_2_-2 (10 nM) for 48 h. After incubation the cells were collected and washed with PBS by centrifugation. Then the cells were lysed with RIPA buffer by triple freezing and thawing, and samples containing 20 μg of total proteins were prepared. The proteins were separated by electrophoresis in 12.5% PAA-gel in denaturing conditions and then were transferred (Semi-dry, Helicon, Russia) on nitrocellulose membrane (Sigma-Aldrich, USA). The P2X4 zone on the blot were revealed using the rabbit anti-P2X4 antibodies (1:200, Abcam plc, Cambridge, UK) and goat anti-rabbit IgG-peroxidase antibodies (1:10000, Sigma-Aldrich, USA), and visualized by enhanced chemiluminescence method using ChemDoc system (Bio-Rad, USA). The β-actin zone was revealed using specific anti-β-actin antibodies (1:1000, Abcam plc, Cambridge, UK) and anti-rabbit IgG-peroxidase antibodies (1:10000, Sigma-Aldrich, USA).

### siRNA transient transfection

siRNA Silencer® Select Pre-Designed siRNA for P2X4, Silencer® Select Negative Control (scrambled siRNA), Opti-MEM® Reduced Serum Medium and Lipofectamine® RNAiMAX Transfection Reagent were purchased in Ambion (USA). Transfection of macrophages with siRNA was performed according to the manufacture’s protocol in 6-well plates containing 1 × 10^6^ cells/well. Briefly, 9 μl of Lipofectamine® RNAiMAX in 150 μl of Opti-MEM was mixed with either 30 pmol P2X4 R siRNA or 30 pmol scrambled siRNA in 150 μl of Opti-MEM and used per one well. The mixture was then incubated for 5 min at room temperature and was added to macrophages with a final siRNA concentration of 25 pmol per well. After 48 h incubation, media were changed and cells were collected for Ca^2+^ imaging. Real-Time PCR was used to confirm P2X4 R knockdown.

### PCR

Total RNA was isolated from 1 × 10^6^ murine macrophages using the TRIZOL reagent according to the manufacturer’s instructions (GIBCOBRL, Gaithersburg, MD). RNA was reverse transcribed into complementary DNA using an MMLV RT kit (Evrogen, Russia). To enrich the P2X4 cDNA the step-out primer was added: 5′ -GACCCTGCTCGTAGTCTTCCACATA-3′. Murine P2X4 and β-actin DNA was amplified using the specific primers: P2X4 F 5′-CAGATCAAGTGGGACTGCAACC-3′ and P2X4 R 5′-ACACGATGATGTCAAAGCGGATG -3′; β-actin F 5′-AGA GGG AAA TCG TGC GTG AC -3′ and β-actin R 5′-CAA TAG TGA TGA CCT GGC CGT -3′. Amplification was performed using a hot start protocol with 45 cycles of the following sequential steps: 96 °C for 15 s, 61 °C for 50 s with RotorGene Q 5plex HRM system (QIAGEN, Germany). PCR products were visualized on a 2% agarose gels using ethidium bromide staining and identified in the expected positions on gels accordingly of their sizes.

### Modeling the mP2X4 receptor-cucumarioside А_2_-2 interaction

Spatial structure model of mouse P2X4 receptor fragment (mP2X4) comprising the extracellular and transmembrane receptor domains (30–354 aa) was generated using the Modeller 9.11 program^30^. The amino acid sequence was obtained from UNIPROT database (ID Q9JJX6). The spatial coordinates of the atomic structure of zfP2X4 in the ATP-bound state (PDB ID 4DW1 at 2.8 Å resolution)^17^, was used as template, RMSD for 324 Cα-atoms of model comparative to prototype was 0.553 Å. For mP2X4 model relaxation, the system was integrated into a palmitoyl oleoyl phosphatidylcholine (POPC) lipid bilayer and solvated in the water box (model Tip3) implicating 0.2 M NaCl presence. Water molecules having the oxygen atoms arranged closer to 3.8 Å of non-hydrogen protein atoms as well as lipid molecules, with an atom closer 1.3 Å to protein non-hydrogen atoms have been removed. Protein-lipid ensemble was modeled using VMD program, and the solvation procedure as well as energy minimizing of the system was carried out in MOE program (CCG)^31^,^32^.

The cucumarioside А_2_-2 spatial structure was constructed using Discovery Studio 3.5 Visualizer program (Accelrus). For optimization of electric properties and a configuration of cucumarioside А_2_-2 molecule a semiempirical quantum chemistry software package MOPAC2009 and semiempirical PM6 method^33^, integrated into package Molecular Docking Server^34^ have been used.

#### Protein-ligand docking

Models of spatial structure of complexes mP2X4 with ATP and cucumarioside А_2_-2 have been constructed via “blind” molecular docking of flexible cucumarioside А_2_-2 structure to rigid extracellular domain of the receptor though the instrumentality of Molecular Docking Server^34^.

#### Molecular dynamics simulation

Computations of molecular dynamics simulation for protein-ligand complexes were performed under conditions of constant pressure, 300 K, and pH 7.0 for 2 ns in an Amber12EHT force field using MOE program (CCG). Prior to molecular dynamic simulations whole system was equilibrated to reduce initial bad contacts. Equilibration consisted in energy minimization of the initial side chains position with fixed backbone atoms, followed by a minimization with restrained carbon alpha atoms and a short molecular dynamics (10 ps). Computer simulation and theoretical studies were performed using cluster CCU “Far Eastern computing resource” FEB RAS (Vladivostok).

### Surface plasmon resonance (SPR)

The study of intermolecular interactions was performed using the optical biosensor Biacore T200 (GE Healthcare, USA), operating on a SPR effect. SPR signals in resonance units were recorded independently in each channel of the biosensor in form sensorgrams showing its changing in time. The following reagents were obtained from GE Healthcare for the operation of SPR biosensor: HBS-N buffer (150 mM NaCl, 10 mM HEPES, pH 7.4), 1-ethyl-3-(3-dimethylaminopropyl)carbodiimide-HCl (EDC), N-hydroxysuccinimide (NHS), 10 mM sodium acetate buffer (pH 4.5). The 3-[(3-Cholamidopropyl)dimethylammonio]−1-propanesulfonate hydrate (CHAPS) were obtained from Sigma Aldrich (USA). Analytical grade NaCl were obtained from local supplier. All experiments were performed at 25 °C using standard optical chip CM5 coated with the layer of carboxymethylated dextran. HBS-N buffer was used as running buffer. Carboxylic groups of dextran were activated by injection the mixture of equal volumes of 0.2 M EDC and 0.05 M NHS for 7 min at a flow rate 5 μL/min and washing with running buffer at the same speed for 1 minute. For SPR studies recombinant mouse P2X4 purinoceptors (sequence positions 55–338aa; the complete extracellular domain) including a N-terminal His-6 tag were expressed in *E. coli* (MyBioSource, Inc., USA). Immobilization of Р2Х4 protein on the surface of the optical chip CM5 was performed by injection of protein solution (20 μg/mL) in 10 mM sodium acetate buffer (pH 4.5) for 20 min at a flow rate 2 μL/min. Remaining activated carboxylic groups of dextran, which did not react with protein, were inactivated during washing procedure with running buffer for 60 min at a flow rate 5 μL/min. Stock solution of the CA_2_-2 was prepared by it dissolving in water at a concentration of 3 mM and was further diluted by HBS-N buffer up to test concentrations. Analysis of CA_2_-2 interactions with the immobilized Р2Х4 receptor was performed by injection of test solutions for 6 min at a flow rate of 10 μL/min. Dissociation of the complexes were recorded for at least 600 sec. The optical chip surface was regenerated by two sequential injection of the regenerating solution (2 M NaCl, 0.4% (m/v) CHAPS) for 17 s at a flow rate of 35 μL/min. Dissociation constant (Kd) of CA_2_-2/Р2Х4 complex was calculated from sensorgram set using software BIAevaluation v.4.1 (GE Healthcare).

### Statistical analysis

Average value, standard error, standard deviation and p-values in all experiments were calculated and plotted on the chart using SigmaPlot 3.02 (Jandel Scientific, San Rafael, CA USA). Statistical difference was evaluated by *t*-test, and results were considered as statistically significant at *p *< 0.05.

## Additional Information

**How to cite this article**: Aminin, D. *et al*. Glycosides from edible sea cucumbers stimulate macrophages via purinergic receptors. *Sci. Rep.* **6**, 39683; doi: 10.1038/srep39683 (2016).

**Publisher's note:** Springer Nature remains neutral with regard to jurisdictional claims in published maps and institutional affiliations.

## Supplementary Material

Supplementary Information

Supplementary Video
