# Supplementary material for: Glycosides from edible sea cucumbers stimulate macrophages via purinergic receptors
Source: Sci Rep. 2016 Dec 22;6:39683. doi: 10.1038/srep39683 (PMC5177912; doi:10.1038/srep39683)
Supplement: Supplementary Information [file srep39683-s1.pdf]

SUPPLEMENTARY INFORMATION

Glycosides from edible sea cucumbers stimulate macrophages *via* purinergic receptors

Dmitry Aminin[1†]*, Evgeny Pislyagin[1†], Maxim Astashev[2], Andrey Es'kov[1], Valery Kozhemyako[1], Sergei Avilov[1], Elena Zelepuga[1], Ekaterina Yurchenko[1], Leonid Kaluzhskiy[3], Emma Kozlovskaya [1], Alexis Ivanov[3] & Valentin Stonik[1†]

[1]G.B. Elyakov Pacific Institute of Bioorganic Chemistry, Far Eastern Branch of the Russian Academy of Sciences, Vladivostok, 690022, Russia.

[2]Institute of Cell Biophysics, Russian Academy of Sciences, Pushchino, Moscow Region 142290, Russia

[3]Institute of Biomedical Chemistry, Moscow, 119121, Russia

[†] These authors contributed equally to this work.

* Corresponding author. E-mail: daminin@piboc.dvo.ru

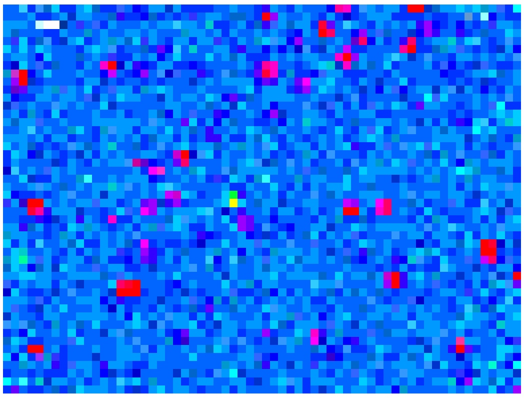

Supplementary Video S1. Calcium imaging in mouse peritoneal macrophages loaded with Fura-2/AM. Increase in $[Ca^{2+}]_i$ in cellular cytoplasm was evoked by successive additions of ATP (100 μM), ATP (100 μM) and CA_2-2 (100 nM). Video was recorded using imaging system based on Zeiss observer Z1 microscope (Zeiss, Oberkochen, Germany), Sutter Lambda DG4 light source (Sutter Instruments, Novato, CA, USA) and Perfusion Fast-Step system SF-77B (Warner Instruments, Hamden, CT, USA). Stopwatch in right upper corner indicates the time of recording and moments of ATP or CA_2-2 application, and washing procedure.

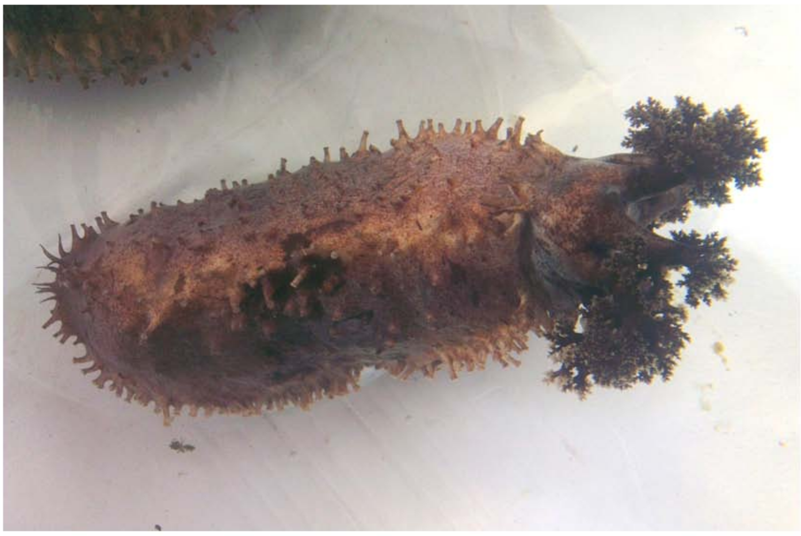

Supplementary Fig. S1. Far-Eastern sea cucumber *Cucumaria japonica*. Photo by Alexander Katantsev (G.B. Elyakov Pacific Institute of Bioorganic Chemistry, Far Eastern Branch of the Russian Academy of Sciences, Vladivostok, Russia)

Supplementary Table S1. List of sea cucumber species, glycosides of which were studied at the G.B. Elyakov Pacific Institute of Bioorganic Chemistry

#	Species	World region
	**Order Aspidochirotida**	
1.	*Holothuria atra*	Tropical part of the Pacific Ocean[33, 42]
2.	*H. arenicola*	Tropical part of the Pacific Ocean[33]<br>Atlantic Ocean, Cuba[34]
3.	*H. cinarescens*	Tropical part of the Pacific Ocean[33]
4.	*H. coluber*	Tropical part of the Pacific Ocean[33]
5.	*H. cubana*	Atlantic Ocean, Cuba[34]
6.	*H. difficilies*	Tropical part of the Pacific Ocean[33]
7.	*H. edulis*	Tropical part of the Pacific Ocean[33,36]
8.	*H. floridana*	Atlantic ocean, Cuba[37,38]
9.	*H. fuscocinerea*	Atlantic ocean, Cuba[33]
10.	*H. gracilis*	Atlantic ocean, Cuba[33]
11.	*H. grisea*	Atlantic ocean, Cuba[34,37]
12.	*H. hilla*	Tropical part of the Pacific Ocean[33]
13.	*H. impatiens*	Tropical part of the Pacific Ocean[33]
14.	*H. leucospilota*	Tropical part of the Pacific Ocean[33]
15.	*H. mexicana*	Atlantic ocean, Cuba[34]
16.	*H. nobilis*	Tropical part of the Pacific Ocean[33]
17.	*H. pervicax*	Tropical part of the Pacific Ocean[33]
18.	*H. polii*	Mediterranean Sea[35]
19.	*H. pulla*	Tropical part of the Pacific Ocean[33]
20.	*H. scabra*	Tropical part of the Pacific Ocean[33]
21.	*H. squamifera*	Tropical part of the Pacific Ocean[49]
22.	*H. surinamensis*	Atlantic Ocean, Cuba[34]
23.	*H. tubulosa*	Mediterranean Sea[35]
24.	*Actinopyga echinites*	Tropical part of the Pacific Ocean[33]
25.	*A. agassizii*	Atlantic Ocean, Cuba[34]
26.	*A. lecanora*	Tropical part of the Pacific Ocean[33]
27.	*A. mauritiana*	Tropical part of the Pacific Ocean[33]
28.	*A. miliaris*	Tropical part of the Pacific Ocean[33]
29.	*A. multifidus*	Atlantic Ocean, Cuba[34]
30.	*Bohadschia argus*	Atlantic Ocean, Cuba[33,52]
31.	*B. marmorata*	Atlantic Ocean, Cuba[33, 52]
32.	*B.tenuissima*	Atlantic Ocean, Cuba[52]
33.	*B. vitiensis*	Atlantic Ocean, Cuba[52]
34.	*Pearsonothuria (Bohadschia) graeffei*	Atlantic Ocean, Cuba[33,44]
35.	*Stichopus chloronotus*	Tropical part of the Pacific Ocean[39-41,45]
36.	*S. japonicus*	Pacific Ocean, Sea of Japan[43,51]
37.	*S. variegates*	Tropical part of the Pacific Ocean[39,40]
38.	*Australostichopus (stichopus) mollis*	Pacific Ocean, New Zealand waters[72,75]
39.	*Thelenota ananas*	Tropical part of the Pacific Ocean[8]
40.	*Pseudostichopus trachus*	Pacific Ocean, Sea of Okhotsk[55,73]
41.	*Synallactes nozawai*	Pacific Ocean, Sea of Japan, deep waters[71]

	**Order Dendochirotida**	
42.	*Actinocucumis typica*	Indian Ocean, Arabian Sea[11,90]
43.	*Eupentacta (Cucumaria) fraudatrix*	Pacific Ocean, Sea of Japan[46,47,57-59, 65, 83-88]
44.	*E. pseudoquiquisemita*	Pacific Ocean, Sea of Okhotsk[54]
45.	*Cladolabes schmeltzii*	Pacific Ocean,Vietnamese waters[91]
46.	*Cucumaria japonica*	Pacific Ocean, Sea of Japan[50, 60,61,63-66,69]
47.	*C. frondosa*	Arctic Ocean, Sea of Barents[63]<br>Atlantic Ocean, Gulf of Maine[70,74,76,78]
48.	*C. koraiensis*	Pacific Ocean, Sea of Okhotsk[67]
49.	*C. Miniata*	Pacific Ocean, Sea of Japan[69]
50.	*C. ochotensis*	Pacific Ocean, Sea of Okhotsk[77,79]
51.	*Dyasmodactyla kurilensis*	Pacific Ocean, Sea of Okhotsk[62]
52.	*Neothynidium magnum*	Pacific Ocean,Vietnamese waters[56]
53.	*Psolus fabricii*	Pacific Ocean, Sea of Okhotsk[48]
54.	*P. eximius*	Pacific Ocean, Sea of Okhotsk[68]
55.	*Pseudocolochirus violaceus*	Pacific Ocean, Vienamese waters[90]
56.	*Staurocucumis liouvillei*	Atlantic Ocean, Antarctic waters[81,82]
57.	*S. turqueti*	Atlantic Ocean, Antarctic waters[89]
	**Order Malpodonia**	
58.	*Paracaudina ronsonetti*	Pacific Ocean, Sea of Japan[53]
	**Order Apodida**	
59.	*Synapta maculate*	Pacific Ocean,Vietnamese waters[80]
	**Order Elasipodida**	
60.	*Achlionice violaescuspidata*	Atlantic Ocean, Antarctic waters[81]
61.	*Kolga hyaline*	Arctic Ocean[92]

33. Elyakov, G.B. et al. Glycosides of marine invertebrates-I. A comparative study of glycoside fraction of Pacific sea cucumbers. *Comp Biochem Physiol* **44B** 325–336 (1973).
34. Elyakov, G.B., Kuznetsova, T.A., Stonik, V.A., Levin, V.S. & Albores R. Glycosides of marine invertebrates-IV. A comparative study of the glycosides from Cuban sublittoral holothurians. *Comp Biochem Physiol* **52B** 413–417 (1975).
35. Silchenko, A.S. et al. Holothurins B_2, B_3 and B_4, new triterpene glycosides from Mediteranean sea cucumbers of the genus Holothuria. *J Nat Prod* **68** 564–567 (2005).
36. Kalinin, V.I. & Stonik, V.A. G1ycosides of marine invertebrates. Structure of ho1othurin A_2 from the holothurian Holothuria edulis. *Khim Prirod Soedin* 215-219 (1982).
37. Oleinikova, G.K. et al. Glycosides of marine invertebrates XV. The new triterpenic glycoside holothurin A_1 from caribbean sea cucumber of the family Holothurioidae. *Khim Prirod Soedin* 464-469 (1982).
38. Kuznetsova, T.A. et al. G1ycosides of marine invertebrates. XIV. Structure of ho1othurin B_1 from the sea cucumber Ho1othuria floridana. *Khim Prirod Soedin* 482-484 (1982).
39. Stonik, V.A., Maltsev. I.I., Ka1inovsky, A.I., Konde, K. & E1yakov, G.B. G1ycosides of marine invertebrates. XI. Two new triterpene glycosides from sea cucumbers belonging

to the family Stichopodidae. *Khim Prirod Soedin* 194-199 (1982).
40. Stonik, V.A., Ma1tsev, I.I., Kalinovsky, A.I. & E1yakov, G.B. G1ycosides of marine invertebrates. XII. Structure of new triterpene oligoglycoside from sea cucumbers belonging to the family Stichopodidae. *Khim Prirod Soedin* 200-204 (1982).
41. Sharipov, V.F., Chumak, A.D., Stonik, V.A. & Elyakov, G.B. G1ycosides of marine invertebrates. X. Structure of stichoposides A and B from holoturian Stichopus chloronotus. *Khim Prirod Soedin* 181-184 (1981).
42. Stonik, V.A. et al. Glycosides of marine invertebrates. VII. Structure of holothurin B from Holothuria atra. *Khim Prirod Soedin* 522-527 (1979).
43. Elyakov, G.B., Maltsev, I.I., Kalinovsky, A.I. & Stonik, V.A. Structure of holotoxin A_1, the main triterpene glycoside from Pacific food holothurian Stichopus japonicus Selenka. *Bioorg Khim* **9** 280-281 (1983)
44. Kalinin, V.I. & Stonik, V.A. G1ycosides of the ho1othurian Bohadschia graeffei. *Khim Prirod Soedin* 789-790 (1982).
45. Maltsev, I.I., Stonik, V.A. & Kalinovksii, A.I. Stichoposide E – a new triterpene glycoside from sea cucumbers belonging to the family Stichopodidae. *Khim Prirod Soedin* 308-312 (1983).
46. Afiyatullov, Sh.Sh., Kalinovsky, A.I. & Stonik, V.A. Structure of cucumariosides C_1 and C_2 – two novel triterpene glycosides from the sea cucumber Cucumaria fraudatrix. *Khim Prirod Soedin* 831-837 (1987).
47. Afiyatullov, Sh.Sh., Tischenko, L.Ja., Stonik, V.A., Kalinovsky, A.I. & Elyakov, G.B. Structure of cucumarioside G_1 – a new triterpene glycoside from the sea cucumber Cucumaria fraudatrix. *Khim Prirod Soedin* 244-248 (1985).
48. Kalinin, V.I., Kalinovsky, A.I. & Stonik, V.A. Psolusoside A – a new triterpene glycoside from the sea cucumber Psolus fabricii. *Khim Prirod Soedin* 212-217 (1985).
49. Ivanova, N.S., Smetanina, O.F. & TA Kuznetsova, T.A. G1ycosides of marine invertebrates. XXVI. Holothurin A from the Pacific Ocean sea cucumber Holothuria squamifera. Isolation of the native aglycone. *Khim Prirod Soedin* 448-451 (1984).
50. Avilov, S.A., Stonik, V.A. & Kalinovsky, A.I. Structures of four new triterpene glycosides from the sea cucumber Cucumaria japonica. *Khim Prirod Soedin* 787-793(1990).
51. Maltsev, I.I., Stonik, V.A., Kalinovsky, A.I. & Elyakov, G.B. Triterpene Glycosides from Sea Cucumber Stichopus japonicus Selenka. *Comp Biochem Physiol* **78B** 421-426 (1984).
52. Antonov, A.S. & Stonik, V.A. Glycosides of sea cucumbers of the genus Bohadschia. *Khim Prirod Soedin* 379-380 (1986)
53. Kalinin, V.I., Maljutin A.N. & Stonik, V.A. Caudinoside A – a new triterpene glycoside from the sea cucumber Paracaudina ransonetii. *Khim Prirod Soedin* 378-379 (1986).
54. Kalinin, V.I., Afiyatullov, Sh.Sh. & Kalinovsky, A.I. Triterpene glycosides of the sea cucumber Eupentacta pseudoquinquesemita. *Khim Prirod Soedin* 221-225 (1988).
55. Kalinin, V.I., Stonik, V.A., Kalinovsky A.I. & Isakov V.V. Structure of pseudostichoposide A – a major triterpene glycoside from the sea cucumber Pseudostichopus trachus. Khim Prirod Soedin 678-684 (1989).
56. Avilov, S.A., Kalinovsky, A.I. & Stonik V.A. New triterpene glycoside from the sea cucumber Neothyonidium magnum. *Khim Prirod Soedin* 53-57 (1990).
57. Avilov, S.A., Kalinin, V.I., Kalinovsky, A.I. & Stonik V.A. Cucumarioside G_2 – a minor triterpene glycoside from the sea cucumber Eupentacta fraudatrix. *Khim Prirod Soedin* 438-439 (1991).
58. Kalinin, V.I. et al. Cucumarioside G_4 – a new triterpene glycoside from the sea cucumber Eupentacta fraudatrix. *Khim Prirod Soedin* 691-694 (1992).

59. Kalinin, V.I., Avilov, S.A., Kalinovsky, A.I. & Stonik V.A. Cucumarioside G_3 – a minor triterpene glycoside from the sea cucumber Eupentacta fraudatrix. *Khim Prirod Soedin* 729-730 (1992).
60. Drozdova, O.A., Avilov, S.A., Kalinovsky, A.I. & Stonik V.A. A new acetylated glycoside from the sea cucumber Cucumaria japonica. *Khim Prirod Soedin* 590-591 (1992).
61. Drozdova, O.A., Avilov, S.A., Kalinovsky, A.I. & Stonik V.A. Minor glycoside from the sea cucumber Cucumaria japonica. *Khim Prirod Soedin* 593 (1992).
62. Avilov, S.A., Kalinovsky, A.I. & Stonik, V.A. Two new triterpene glycosides from the sea cucumber Duasmodactyla kurilensis. *Khim Prirod Soedin* 221-226 (1991).
63. Avilov, S.A. et al. Triterpene glycosides from the sea cucumber Cucumaria frondosa. *Khim Prirod Soedin* 260-263 (1993)
64. Drozdova, O.A et al. New glycosides from the sea cucumber Cucumaria japonica. *Khim Prirod Soedin* 242-248 (1993).
65. Avilov, S.A., Kalinin, V.I., Makarieva, T.N., Stonik, V.A. & Kalinovsky, A.I. Structure of cucumarioside G_2, a novel nonholostane glycoside from the sea cucumber Eupentacta fraudatrix. *J Nat Prod* **57** 1166-1171 (1994).
66. Avilov, S.A., Tischenko, L.Ja. & Stonik, V.A. Structure of cucumarioside A_2-2 – a triterpene glycoside from the sea cucumber Cucumaria japonica. *Khim Prirod Soedin* 799-800 (1984).
67. Avilov, S.A. et al. Koreoside A, a new nonholostane triterpene glycoside from the sea cucumber Cucumaria koraiensis. *J Nat Prod* **60** 808-810 (1997).
68. Kalinin, V.I. et al. Structure of eximisoside A, a novel triterpene glycoside from the Far-Eastern sea cucumber Psolus eximius. *J Nat Prod* **60** 817-819 (1997).
69. Drozdova, O.A. et al. Cytotoxic triterpene glycosides from Far-Eastern sea cucumbers belonging to the genus Cucumaria. *Liebigs Ann* 2351-2356 (1997).
70. Avilov, S.A. et al. Frondoside C, a new nonholostane triterpene glycoside from the sea cucumber Cucumaria frondosa: structure and cytotoxicity of its desulfated derivative. *Can J Chem* **76** 137-141 (1998).
71. Silchenko, A.S. et al. Triterpene glycosides from the deep-water North-Pacific sea cucumber Synallactes nozawai Mitsukuri. *J Nat Prod* **65** 1802-1808 (2002).
72. Moraes G. et al. Structure of major triterpene glycoside from the sea cucumber Stichopus mollis and evidence to reclassify this species into the new genus Australostichopus. *Biochem Syst Ecol* **32** 637-650 (2004).
73. Silchenko, A.S. et al. Pseudostichoposide B – new triterpene glycoside with unprecedent type of sulfatation from deep-water North-Pacific sea cucumber Pseudostichopus trachus. *Nat Prod Res* **18** 565-570 (2004).
74. Silchenko, A.S. et al. Glycosides from the sea cucumber Cucumaria frondosa III. Structure of frondosides A_2-1, A_2-2, A_2-3 and A_2-6, four new minor monosulfated triterpene glycosides. *Can J Chem* **83** 21-27 (2005).
75. Moraes, G. et al. Mollisosides A, B_1 and B_2: Minor triterpene glycosides from the New Zealand and South Australian sea cucumber Australostichopus mollis. *J Nat Prod* **68** 842-847 (2005).
76. Silchenko, A.S. et al. Glycosides from the sea cucumber Cucumaria frondosa. IV. Structure of frondosides A_2-4, A_2-7, and A_2-8, three new minor monosulfaed triterpene glycosides. *Can J Chem* **83** 2120-2126 (2005).
77. Silchenko, A.S. et al. Monosulfated triterpene glycosides from Cucumaria okhotensis Levin et Stepanov, a new species of sea cucumbers from sea of Okhotsk. *Bioorg Khim* **33**, 81-90 (2007).
78. Silchenko, A.S. et al. Glycosides from the North Atlantic sea cucumber Cucumaria frondosa V – Structures of five new minor trisulfated triterpene oligoglycosides, frondosides A_7-1, A_7-3, A_7-4, and isofrondoside C. *Can J Chem* **85** 626-636 (2007).

79. Silchenko, A.S. et al. Constituents of the Sea Cucumber Cucumaria okhotensis. Structures of okhotosides B_1–B_3 and cytotoxic activities of some glycosides from this species. *J Nat Prod* **71** 351-356 (2008).
80. Avilov, et al. Synaptosides A and A_1, Two triterpene glycosides from the sea cucumber Synapta maculata containing 3-O-methylglucuronic acid and their cytotoxic activity against tumor cells. *J Nat Prod* **71** 525-531 (2008).
81. Antonov, A.S. et al. Triterpene glycosides from Antarctic sea cucumbers I. Structure of liouvillosides A_1, A_2, A_3, B_1 and B_2 from the sea cucumber Staurocucumis liouvillei, new procedure for separation of highly polar glycoside fractions and taxonomic revision. *J Nat Prod* **71** 1677-1685 (2008).
82. Antonov, A.S. et al. Triterpene glycosides from Antarctic sea cucumbers. III: Structures of liouvillosides A_4 and A_5, two minor disulphated tetraosides containing 3-O-methylquinovose as terminal monosaccharide units from the sea cucumber Staurocucumis liouvillei (Vaney). *Nat Prod Res* **25** 1324-1333 (2011).
83. Silchenko, et al. Triterpene glycosides from the sea cucumber Eupentacta fraudatrix. Structure and cytotoxic action of cucumariosides A_2, A_7, A_9, A_{10}, A_{11}, A_{13} and A_{14}, seven new minor non-sulated tetraosides and an aglycone with an uncommon 18-hydroxy group. *Nat Prod Commun* **7** 845-852 (2012).
84. Silchenko, A.S. et al. Triterpene glycosides from the sea cucumber Eupentacta fraudatrix. Structure and biological actions of cucumariosides A_1, A_3, A_4, A_5, A_6, A_{12} and A_{15}, seven new minor non-sulfated tetraosides and unprecedented 25-keto,25-norholostane aglycone. *Nat Prod Commun* **7** 517-525 (2012).
85. Silchenko, at al. Structures and cytotoxic properties of cucumariosides H_2, H_3 and H_4 from the sea cucumber Eupentacta fraudatrix. *Nat Prod Res* **26** 1765-1774 (2012).
86. Silchenko, at al. Triterpene glycosides from sea cucumber Eupentacta fraudatrix. Structure and biological activity of cucumariosides B_1 and B_2, two new minor non-sulfated unprecedented triosides. *Nat Prod Commun* **7** 1157-1162 (2012).
87. Silchenko, at al. 3β-O-Glycosylated 16β-acetoxy-9β-H-lanosta-7,24-diene-3β,18,20β-triol, an intermediate metabolite from the sea cucumber Eupentacta fraudatrix and its biosynthetic significance. *Biochem Syst Ecol* **44** 53-60 (2012).
88. Silchenko, A.S. et al. Structure of cucumariosides H_5, H_6, H_7 and H_8. Glycosides from the sea cucumber Eupentacta fraudatrix and unprecedented aglycone with 16,22-epoxy-group. *Nat Prod Commun* **6** 1075-1082 (2011).
89. Silchenko, et al. Triterpene glycosides from Antarctic sea cucumbers IV. Turquetoside A, a 3-O-methylquinovose containing disulfated tetraoside from the sea cucumber Staurocucumis turqueti (Vaney, 1906) (=Cucumaria spatha). *Biochem Syst Ecol* **51** 45-49 (2013).
90. Silchenko, A.S. et al. Structures and biological activities of typicosideds A_1, A_2, B_1, C_1 and C_2, triterpene glycosides from the sea cucumbers Actinocucumis typica. *Nat Prod Commun* **8** 301-310 (2013).
91. Silchenko, A.S. et al. Structure and biological action of cladolosides B_1, B_2, C, C_1, C_2 and D, six new triterpene glycosides from the sea cucumber Cladolabes schmeltzii. *Nat Prod Commun* **8** 1527-1534 (2013).
92. Silchenko, A.S. et al. Kolgaosides A and B, two new triterpene glycosides from the Arctic deep water sea cucumber Kolga hyalina (Elasipodida: Elpidiidae). *Nat Prod Commun* **9** 1259-1264 (2014).

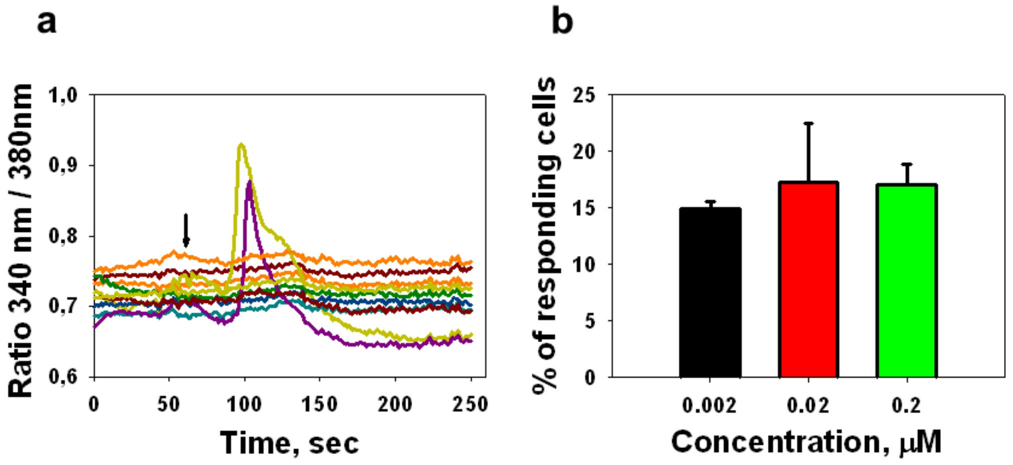

Supplementary Fig. S2. CA_2-2 stimulates part of total fraction of macrophages. Increases in $[Ca^{2+}]_i$ in mouse peritoneal macrophages evoked by CA_2-2 (20 nM) measured with Fura-2/AM (**a**) and the number of responding cells to CA_2-2 application in dependence of glycoside concentration (**b**) in cell monolayer. Arrow indicates the time of CA_2-2 application; each colorful line indicates the intracellular Ca^{2+} level in single cell recorded over time.

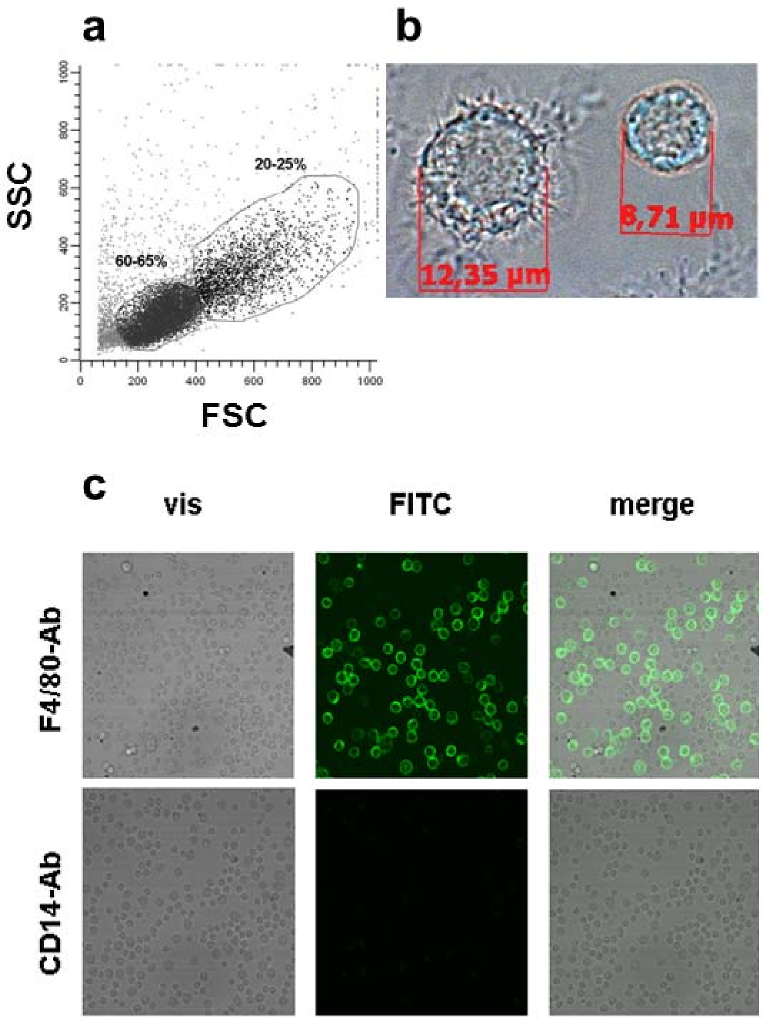

Supplementary Fig. S3. F4/80+ cells represent large macrophages with greater granularity and rougher surface. Determination of geometrical parameters of cells in BALB/c mouse peritoneal macrophage sub-populations by flow cytometry (**a**) and microscopy (**b**). Immunocytochemical staining of mouse peritoneal macrophage population using antibodies to surface markers of monocytes (CD14) and mature macrophages (F4/80) and confocal microscopy (**c**). Secondary antibodies are conjugated with FITC (green color).

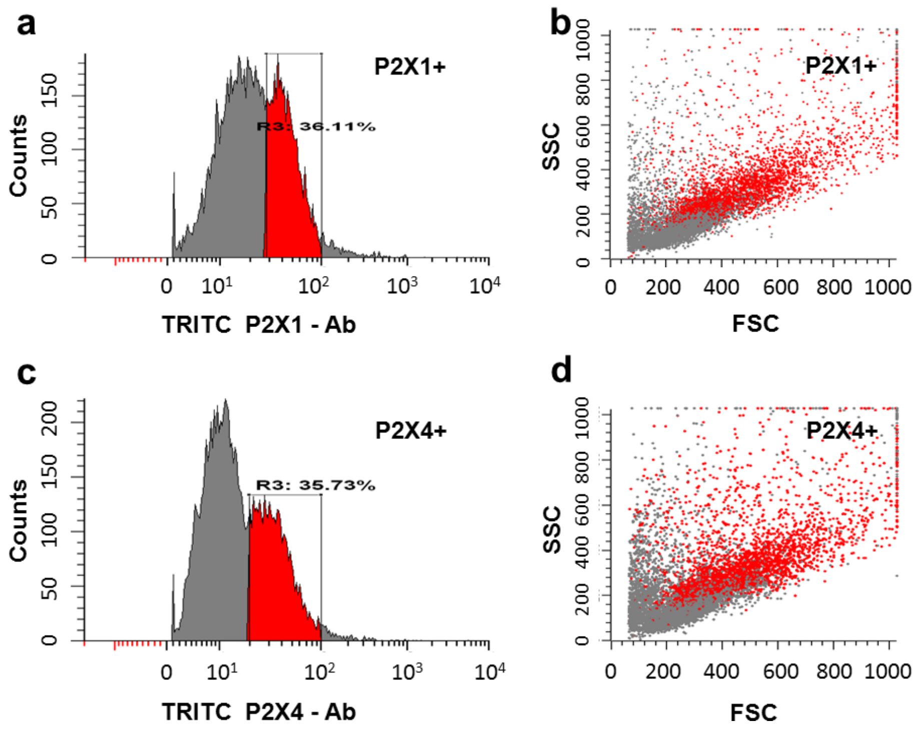

Supplementary Fig. S4. Distribution and quantification of P2X1+ and P2X4+ positive cells in population of BALB/c mouse peritoneal macrophages. Cell analysis was performed by flow cytometry and displayed as histogram (**a,c**) and dotogram (**b,d**). Secondary antibodies for receptor of P2X family detection were conjugated with TRITC (red color)

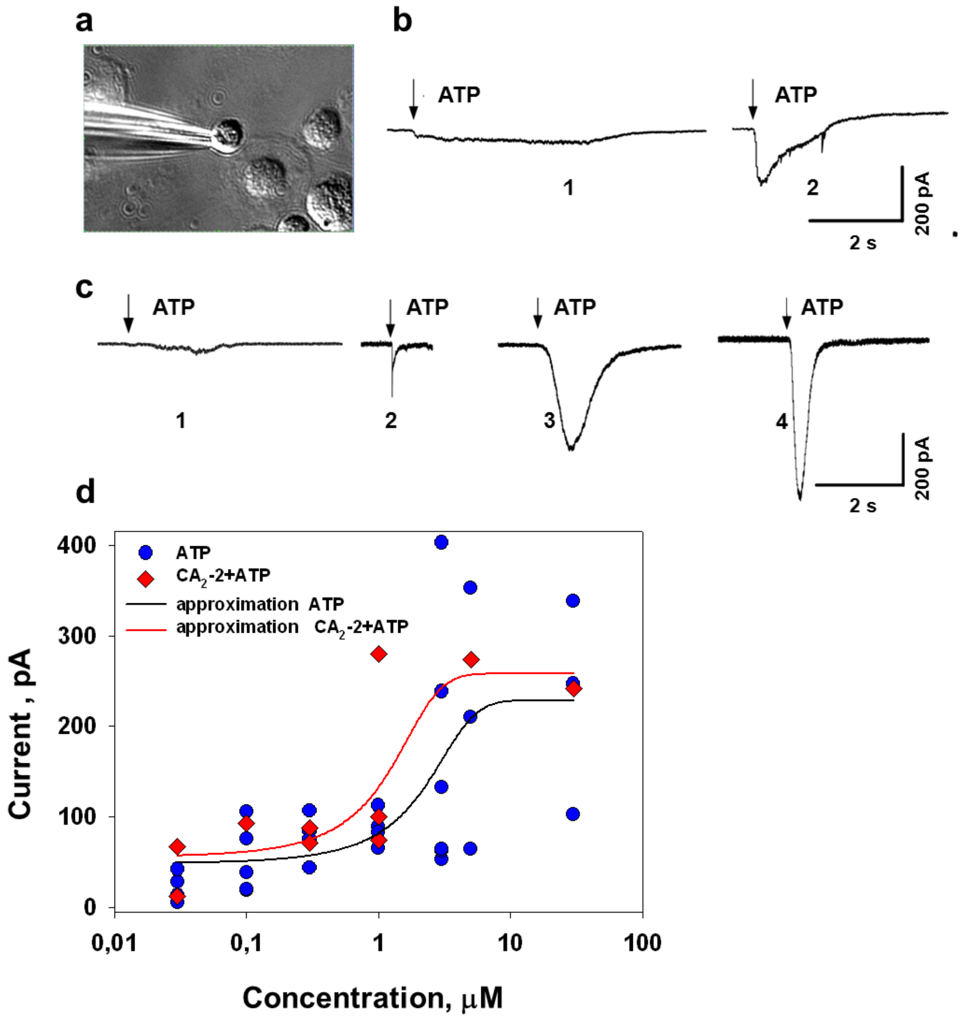

Supplementary Fig. S5. Electrophysiological properties of macrophages assessed by whole-cell patch-clamp recordings. a, Image of a patch pipette attached to the membrane of a cultured mouse peritoneal macrophage; **b,** Records of incoming Ca^{2+} current in macrophage evoked by ATP in concentration 100 µM (1) and 1 mM (2) without pre-incubation with apyrase; **c**, Influence of ATP at concentration of 0.03 µM (1), 0.3 µM (2), 3 µM (4) and 5 µM (4) upon incoming Ca^{2+} current in macrophage pre-incubated with apyrase, 2.0 U/ml. **d,** Dependence of the amplitude of Ca^{2+} current responses of mouse peritoneal macrophages on the concentration of applied ATP in the absence (●) or presence (◆) of CA_2-2, 100 nM.

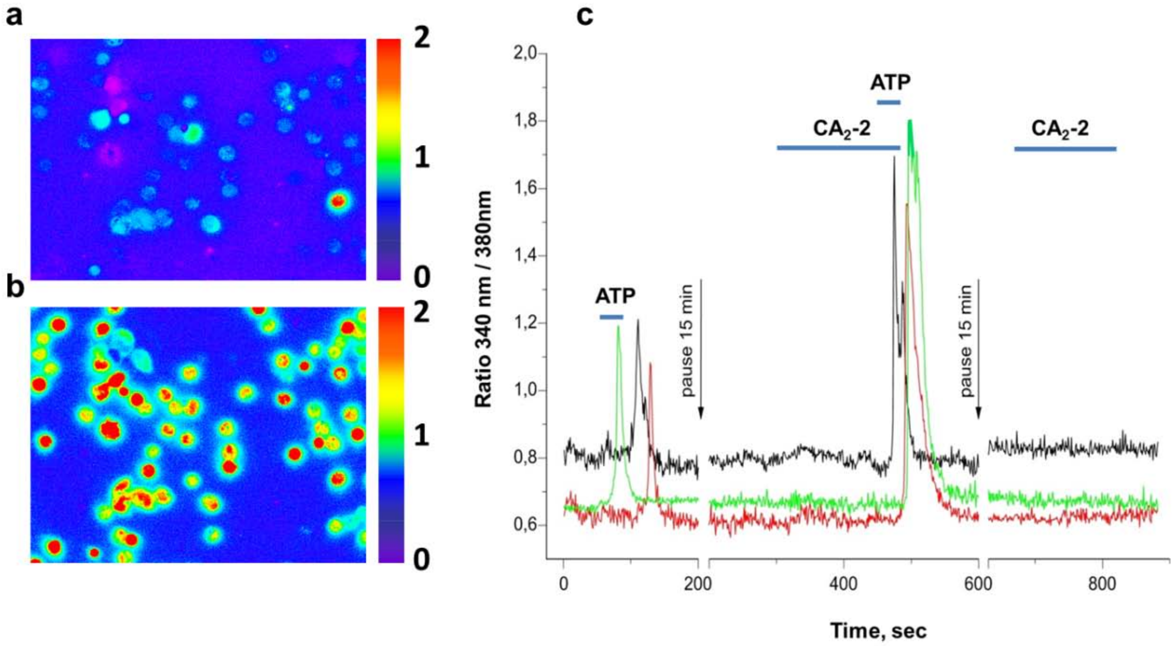

Supplementary Fig. S6. **CA_2-2 increases the amplitude of Ca^{2+} response in macrophages elicited by ATP.** Pseudo-colored Ca^{2+} imaging of macrophages loaded with Fura-2/AM showing cells before stimulation (**a**) and at the peak of the response to 3 μM iomomycin stimulus as a positive control (**b**). **c**, 10 μM ATP was applied to mouse macrophages. After washing procedure cells were pre-treated with 300 nM CA_2-2 during 2 min followed by next 10 μM ATP application. Then cells were washed up with buffer solution and 300 nM CA_2-2 was added again to incubation chamber. Ca^{2+}-imaging technique was used to detect $[Ca^{2+}]_i$ in macrophages; each colorful line indicates the intracellular Ca^{2+} level in single cell recorded over time. Macrophages were initially preincubated with 2.0 U/ml apyrase.

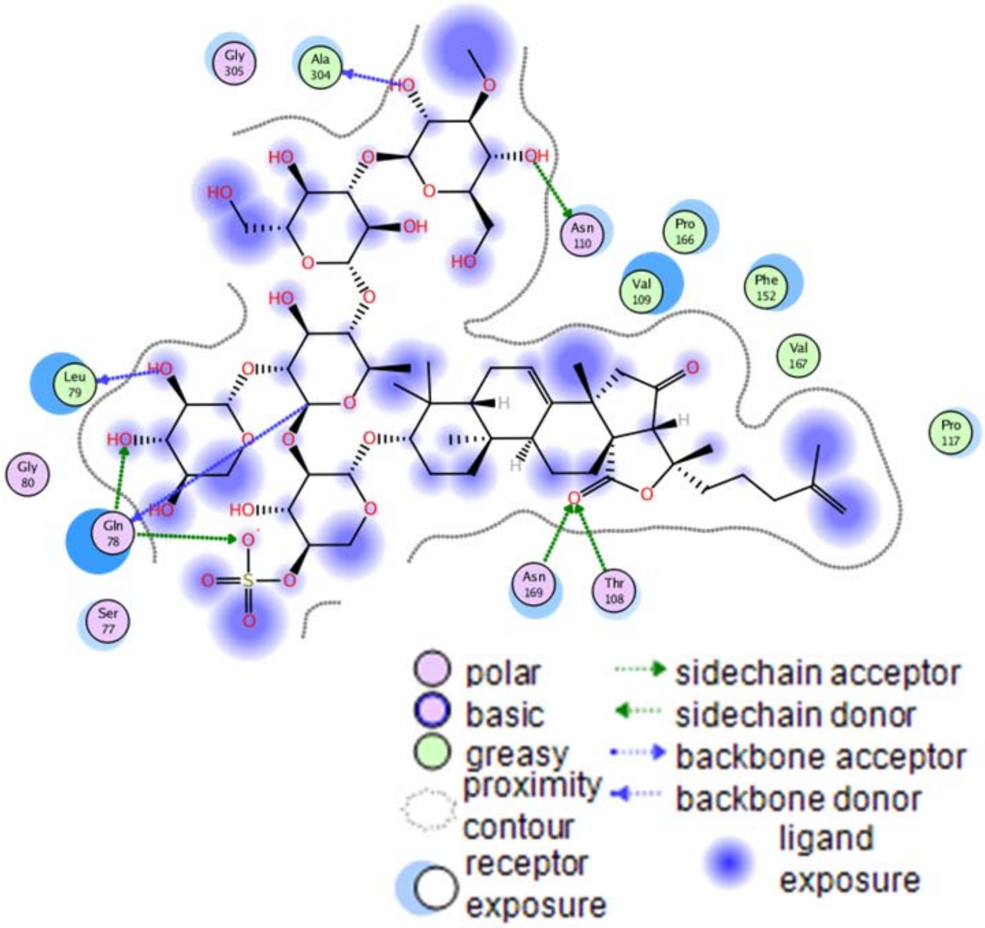

Supplementary Fig. S**7. 2D diagram of CA$_2$-2 contacts with binding site on the receptor mP2X4**. Presentation is performed with the Ligand interaction suite of MOE program.

Supplementary Table S2. Parameters of CA_2-2 binding with P2X4 receptor.

CA_2-2 atoms	mP2X4 residues	ΔG (kcal/mol)
C1	Gln78 H – backbone donor	-0.7
O62	Leu79 H – backbone donor	-0.5
O85	Asn110 H – sidechain donor	-3.0
O88	Ala304 H – backbone donor	-0.7
O20	Gln78 H – sidechain acceptor	-4.3
O48	Thr108 H – sidechain acceptor	-2.4
O48	Asn169 H – sidechain acceptor	-1.0
O63	Gln78 H – sidechain acceptor	-1.1
		**Sum -13.7**

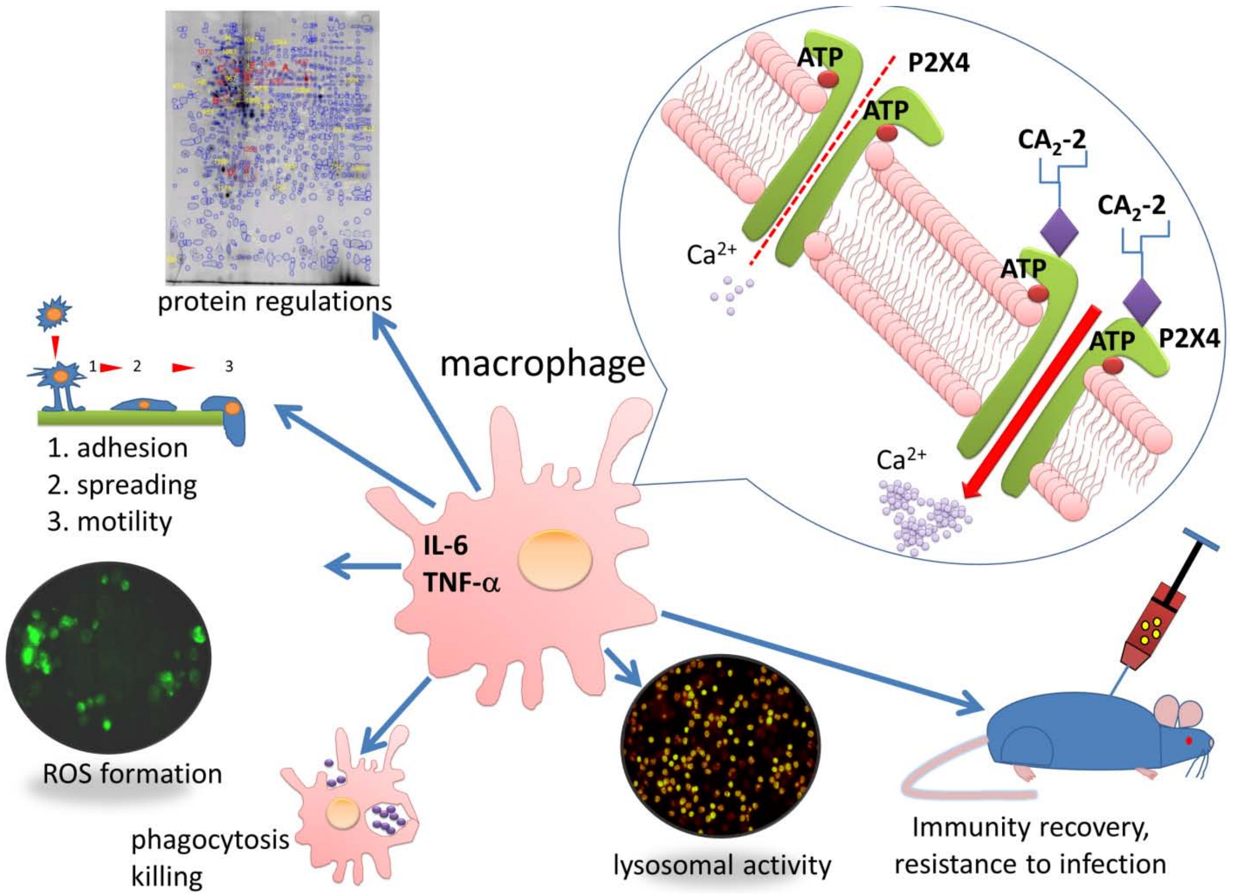

Supplementary Fig. S8. Scheme of CA_2-2 interaction with macrophages. CA_2-2 can act as a regulator which able to modulate purinergic receptor activation by extracellular ATP and provide an increase in Ca^{2+} conductivity of macrophage membrane. Such interaction may trigger an activation of Ca^{2+}-signaling pathway that initiates the amplification of expression of certain intracellular target proteins involved in key stages of immune cell physiology. This leads to increasing in macrophage adhesion, spreading and motility, ROS formation, rise in lysosomal activity, phagocytosis, elevated synthesis of some (IL-6, TNF-α) cytokines and pathogenic microorganism killing. Ultimately, an activation of cellular immunity and magnification of the organism resistance to various opportunistic infections is appeared under glycoside action. In case of chronic inflammation accompanied with P2X receptors inactivation the original immune response is restored and organism comes out of a chronic inflammation condition after CA_2-2 administration.
